# Ethical behaviors by leaders act as a stimulant to the wellbeing of employees by restraining workplace embitterment

**DOI:** 10.3389/fpubh.2022.974642

**Published:** 2022-09-29

**Authors:** Ammara Saleem, Mohsin Bashir, Muhammad Abrar

**Affiliations:** Lyallpur Business School, Government College University Faisalabad, Faisalabad, Pakistan

**Keywords:** ethical leadership behavior, employee emotion, workplace embitterment, employee wellbeing, ethical leadership

## Abstract

Prior studies have revealed that leaders' ethical behaviors significantly influence employees' wellbeing. However, it's unclear how to increase the positive impact of leaders' ethical behaviors on employees' wellbeing by overseeing the negative workplace emotion. So, this study examines the salient concern of leaders' ethical behaviors that affect employees' negative emotions (workplace embitterment) and, consequently, their wellbeing according to appraisal theories of emotions. The study also investigates the active role of followers' core self-evaluation in moderating the impact of leaders' ethical behaviors on followers' emotions and wellbeing *via* the mediational chain. Data is collected in two-time intervals with 6 weeks interims through a structured questionnaire from 398 academics of public sector universities in Pakistan. The structured equation modeling and Process Macro 2017 are the tools for data analysis. Findings of this study show that (1) ethical behaviors by leaders have a negative impact on employee workplace embitterment, (2) workplace embitterment completely mediates the association between ethical behaviors of leaders and employee wellbeing, and (3) when leaders do not exhibit ethical behaviors, workplace embitterment is lessened showing high core self-evaluations by employees. In addition, the study findings also reveal that employees' core self-evaluation moderates the effect of leaders' ethical behaviors through workplace embitterment. This study validates the significant role of a leader's ethical behaviors in nourishing employee wellbeing by preventing negative emotions. The study is also significant as it examines how followers' attribute core self-evaluation: (1) can be a substitute for leaders' ethical behaviors and (2) can actively modify the effect of leaders' ethical behaviors on followers' negative emotions and then wellbeing. The study also discussed its contributions in theory and to organizations.

## Introduction

The survival and advancement of organizations worldwide depend on their employees' wellbeing (1, 2). Employee's poor wellbeing concerns are increasing and extending from the individual to organisational to social levels owning to its inauspicious upshots. Employee wellbeing issues in organizations ascend due to hitches, such as stress, unfairness, and bullying (3–5). However, an employee's wellbeing is snagged by negative emotions (6), such as workplace embitterment (feeling of unfairness and humiliation) that develop owing to numerous organisational events, decisions, and leaders' behaviors (4). Employees' emotions are responses followed by copious events encountered in their relationship with leaders, others, and the organisation's environment (7); they value employee wellbeing. Appraisal theorists of emotions divulge that employee emotions nourish and intensify when an event or situation is important to him [as cited in (8)].

Generally, events in organizations are initiated by leaders responsible for administration and decision-making (1). Those events escorted by injustice or controlled supervision instigate embitterment in employees and eventually influence their wellbeing (1, 9). The emergent research on ethical leadership delineates it as a leader's mechanism that governs employees' emotions either negatively or positively, depending on the genuine leader's ethical practices (5, 7). The relationship between (unethical) behavior and employee wellbeing is convoluted and complex; appraisal theorists contend that, among other processes, the impact of unethical organizational practices on embitterment negatively impacts employee wellbeing (7, 10). Giacalone and Promislo urge that organizations at effective monitoring of low ethical behavior may slacken the emergence of negative emotions and thus advance employee wellbeing (10).

Researchers have explicitly ascertained the significance of leaders' ethical practices, such as fairness, integrity, care for others, and role clarification, for managing low ethical behavior in organizations (5, 7, 11). The majority of this research, which employed the Brown et al. (12) scale and was done in the West, had limited details on South Asian territory. There are eight nations in South Asia, and although sharing borders with Iran and China, Pakistan's culture is distinct and hasn't often been questioned in the literature. Additionally, recent corporate outrages and literature highlight the growing issue of unethical behaviors in organizations of a collectivist culture where we feel obligated to favour our close ones and, developing countries that have scarce resources and poor transparent systems (13, 14). However, ethical leadership is seldom investigated Asian and developing countries, particularly in educational settings (15), significantly influencing academics' emotions and wellbeing. Academics are role models for their students who contribute vigorously to developing moral values and ethics and shaping the character of their students. Researchers have emphasized that negative academic emotions interrupt students' education and cut back the excellence of research work in universities (15). Hence, this study is intended to investigate why and how leaders' ethical behaviors nourish employee wellbeing of academicians of public sector universities of Pakistan by restraining workplace negative emotion, i.e., workplace embitterment.

Keeping into consideration the research question, the first objective of this study is to examine the emotional reaction (workplace embitterment) of followers in response to a leader's ethical behaviors (LEBs) in the relational perspective, i.e., affective events theory (AET). The prior studies that investigated the relationship between LEBs and employee outcomes were based on exchange and identity perspectives (16). Affective events theory explains a strong association between appraising a specific event and the emergence of a specific emotion (8). AET accentuates that positive events develop positive emotions and adverse events advance negative emotions.

The study's second objective is to investigate how negative emotional experiences function as a mediating mechanism for ethical leadership behaviors prioritising employee wellbeing from a relational perspective. Most studies have emphasized the investigation of positive mediating mechanisms, such as employee engagement, LMX, and perceived organizational support, in the leaders' ethical behaviors–employee wellbeing relationship (17–19). Few researchers examined how ethical leadership behaviors indirectly affect employee wellbeing through emotional responses, particularly negative emotions in the observed relationships (20, 21). Leaders' ethical behaviors help their followers cultivate their wellbeing through effective interactions that restrain their negative emotions. Investigating this process allows the management to comprehend the routine yet serious impediments to employee wellbeing.

As the perception of employees about ethical behaviors shown by their leaders varies, the employee may or may not have the presence of LEBs, which influence their emotional experience accordingly. So, another objective is to assess the effectiveness of followers' core self-evaluation (CSE) in managing the association concerning LEBs and workplace embitterment (20). This study considers core self-evaluation as a substitute for a leader's ethical behaviors, which makes either leadership redundant or minimises followers' dependency on the leader. This study highlights its significance from the following contributions.

First and foremost, this study is significant as it expands the literature on employee wellbeing by both organisational factors, i.e., leaders' ethical behaviors and individual factors, i.e., followers' traits such as core self-evaluation. The study emphasises leaders' ethical behaviors in developing countries and a collectivist culture wherein diminishing workplace embitterment and fostering employee wellbeing is mandatory (d). It helps to establish the rational relationship between ethical leadership and negative employee emotions, i.e., workplace embitterment. It also posits the significance of leaders' ethical behaviors in managing negative emotions and promoting employee wellbeing by spotting the logical connexions between LEBs, employees' negative emotions and wellbeing.

Second, the study adds to the literature on workplace embitterment which is limited, particularly in the perspective of a leader's behaviors and leadership process. This study also investigates how leaders' ethical behaviors as a contextual variable are related to employees' negative emotions (workplace embitterment). The present investigation highlights the critical role of leaders' ethical behaviors in stimulating employee wellbeing by preventing or reducing embitterment.

Third, the study contributes to an emergent research area involved with improving employee wellbeing where leaders' ethical behaviors are either missing or limited by examining the role of followers' traits, such as core self-evaluation as a moderator, which may swap leaders' ethical behaviors. The findings of this study emphasise the effectiveness of followers' traits, i.e., core self-evaluation, in developing employee wellbeing by managing their negative emotions (workplace embitterment) in the absence of leaders' ethical behaviors. Thus, the study findings suggest followers' CSE is a swap of the leaders' ethical behaviors to foster employee wellbeing.

**Figure 1:**
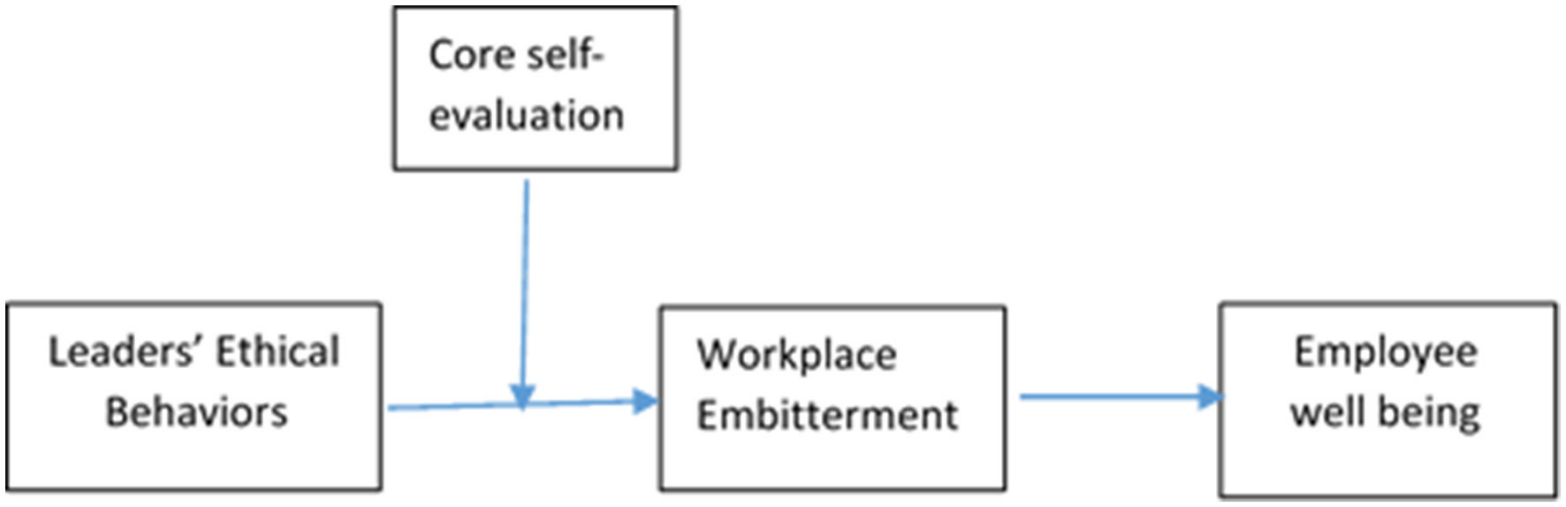
Theoretical model.

Likewise, with theoretical contributions, this study provides some practical benefits to both employees and organizations. This study provides the opportunity for the management to understand how leaders' ethical behaviors improve employees' wellbeing by preventing them from being embittered. Hence, it suggests a guideline for longitudinal tracking of changes in the workplace that encourage leaders to behave ethically, which uplift employees' wellbeing by avoiding negative emotional experiences. In summary, our research has two main objectives. The first is to investigate the possibility of workplace embitterment serving as a mediating mechanism between leaders' ethical behaviors and employees' well-being, as was mentioned before. The second is a test of moderating the impact of the followers' core self-evaluation on the relationship between leaders' ethical behaviors and employee workplace embitterment. In order to evaluate research theories empirically in the context of Pakistan, this study used two-wave data. In Figure 1, the research model is shown.

## Theoretical and hypotheses support

Affective Events Theory (AET) contends that workplace events trigger workplace emotions (22). Events and their frequency determine the intensity of emotions and behaviors at work. According to AET, employees' exposure to negative and unjust events instigate embitterment and thus diminishes their wellbeing (23). Moreover, Lazarus et al. (24) contend that AET suggests that emotional reactions emerge in response to the arousal of emotions that further govern employee behavior. Thus, employees' perception of leaders' behaviors determines emotions or moods and then behaviors. In sum, AET conveys two main features. First, emotions regulate employees' behaviors. AET exhibits how workplace provocations and pleasures affect employees' emotions and wellbeing. Second, leaders' behaviors are significant for employees' emotions and should not be unheeded.

### Leader's ethical behaviors and employee wellbeing

A leader's ethical behavior is operationalised as behaviors that serve as an informal but coherent leadership role in organizations. The essential behaviors such as fairness, honesty, justice, respect, care for others, and building a community for ethical leadership are part of ethical behaviors (25, 26). Brown and Travino defines ethical leadership and talks about behaviors based on trustworthiness, charisma, integrity, feeling of care for their employees, and fairness (11). They discussed that ethical leaders should have the qualities of a moral person and moral manager. The leader as a moral person embodies individual attributes, such as honesty and integrity.

In contrast, a moral manager is a person who is an honest person that takes fair and genuine decisions both in their professional and private life. The dimension of a moral manager discusses a leader's hands-on and active steps to develop an ethical work environment by demonstrating ethical guidelines, being a role model, and keeping ethics on the top priority of the organisational agenda (11). Watts conceptualises ethical leadership (EL) as “Ethical leadership is coordinated by regard for moral convictions and values and keeps up the dignity and privileges of others” (27). A more comprehensive description of EL has been presented: “Ethical leadership conduct manages how leaders utilise their managerial power and leadership role to empower and advance ethical standards and ethical behaviors in the organizations” (11).

As given in the literature, LEBs have a favourable influence on employee attitudes and behaviors, including job performance (28), employee commitment and job satisfaction (29), and employee creativity (30), according to several research works on leaders' ethical behaviors (31, 32). These studies examined this relationship in social exchange and learning perspectives. From the social exchange perspective, employees select their actions based on their relationship with their leaders. Ethical leaders caring, fair, and concerned around their employees can gain their trust and devotion. Employees feel obligated to compensate their leaders through proactive, constructive behaviors and suggestions. In the perspective of social learning, leaders having ethical values are models and mentors for their followers (11). Leaders exhibiting ethical behaviors are charitably spurred and likely to require activities against unfairness and untrustworthy behavior (11). Therefore, employees consider such leaders as their role models.

Employees' wellbeing is a universal concept and has developed over time, and the clear definition is still ambiguous (33). Today, work is still considered an integral part of one's life, and various events and practices at the workplace exert direct and indirect influence on employee wellbeing. The concept of employee wellbeing is different from individual general wellbeing. “Everyone understands the meaning, but nobody can give a precise definition” is how one person described employee wellbeing.

In the literature, psychological wellbeing, subjective wellbeing, or employee satisfaction are used to explain employee wellbeing (21). Subjective wellbeing is explained as one's experience and the overall evaluation of the quality regarding different life aspects, such as personal achievements and social standings, by defined criteria or standards (34). Psychological wellbeing refers to the great condition of mental capacities and the satisfaction of individual potential. Researchers have explained that psychological wellbeing has six dimensions “self-acceptance, personal growth, purpose in life, positive relations with others, environmental mastery, and autonomy” (35). Undoubtedly, subjective and psychological wellbeing concepts are different, but studies have found and discussed some relatedness (36). Chen et al. proposed an integrative approach for assessing employee wellbeing by combining subjective and psychological wellbeing (21, 36). Later, researchers of wellbeing highlighted a missed but essential component of employee wellbeing, i.e., the works-related effect, which is named workplace wellbeing (37). In contemporary societies, work and family are inseparable aspects of one's life. In view of this holistic consideration (33), suggested and developed a measure comprising three components: life, workplace, and psychological wellbeing to assess employee wellbeing. The relationship between leaders' ethical behaviors and employee wellbeing has been studied in several research works [such as (38)]; however, the studies that looked at the different aspects of employee wellbeing about leaders' ethical behaviors are few.

Prior research has shown that both physical and psychological work conditions have an impact on an employee's wellbeing (39). The ethical conduct of leaders is one of the most important psychological workplace factors that affect employee wellbeing (20). Additionally, Schwepker et al. talked about the critical role that ethical leaders have in fostering and strengthening employee wellness through fair treatment, the elimination of disparities, equitable power-sharing, and the provision of ethical standards to their followers (9). Inceoglu et al. also investigated how leaders' ethical behaviors can significantly enhance employee wellbeing, which could benefit both employees and organizations (40).

In today's competitive work environment, the role of leaders' ethical behaviors has gained prominent attention from researchers due to its unique features, such as fairness, behavioral integrity, power sharing, role clarity, concern for employees, and ethical guidelines. Ethical leaders fairly deal and collaborate with their followers for their best interest (13). Such an ethical work climate and ethical relational interaction of followers with their leaders enhance their experience and perception of improved wellbeing (41).

### Ethical behaviors of leader and workplace embitterment

Workplace embitterment is a negative emotion that emerges in response to various organisational destructive events specifically associated with leaders. Linden (24) has explained it as a feeling (emotion) encompassing lasting feelings of being disenchanted, insulted, and vengeful, however, helpless. Those who are embittered perceive that they are treated unjustly, unreasonably, and unfairly; they show a longing for revenge against the individual responsible for their contrary and negative state, yet they reject help from others (42). It can result from a single, extremely intense incident or a string of related life events, according to Sensky (43) and Carter (4). The hallmark of workplace embitterment is that it may potentially arise from an experience that is personally seen as being unreasonable and unfair (43). According to previous studies, organisational injustice, social injustice, abuse of basic principles, mistreatment, and controlled supervision are the key contributing factors to workplace embitterment (43, 44). The absence of leaders' characteristics of fairness, integrity, concern for people, and power sharing in their behaviors are the stimuli of unjust and humiliating events that employees appraise unfavourably, and embitterment may emerge (45).

Employees consistently experience negative emotions at the workplace for various reasons, such as ineffective leader behaviors, strategic unfair decisions, humiliation, and bullying (4, 5, 19). Persistent negative and stressful feelings at the workplace are predominant and have hostile upshots (5, 19). AET states that employees' emotions are extracted from the appraisal of their workplace events. When employees perceive their leaders as less fair, dishonest, less employee-oriented, etc., they appraise such practices negatively, and consequently, negative emotions emerge. According to Velez and Neves, leaders in organizations are uniquely positioned to stimulate an emotional response in followers (20).

A study by Michailidis and Cropley (19) investigated that leaders' practices such as being unfair, task-oriented, controlled, and less trustworthy result in embitterment. Some researchers have discussed that the employee appraisal of his leader's ethical behavior develops or prevents negative feelings as embitterment [e.g., (5, 45)]. Michailidis and Cropley have studied the contributing factors of workplace embitterment by longitudinal design and found that out of four factors of justice, three factors, distributive, informational, and interactional justice, are leader related and are more significant in the development of embitterment emotion than organisational justice which is structurally related (19). In short, a leader's ethical behaviors can reduce employees' embitterment. So, through the lens of effective event theory, we can hypothesise that ethical behaviors of leaders significantly and negatively influence employees' workplace embitterment.

**Hypothesis 1:** Perceived leaders' ethical behaviors will lower employee exposure to workplace embitterment.

### Workplace embitterment as a mediator

The emotional experience and reaction of employees in leader-member interaction is an extensive area of research (5, 46) over the last decades due to its vital role in the leadership process (47) and its ultimate influence on employees' outcomes and wellbeing (1, 5). Workplace embitterment and employee wellbeing relationship may be described by the Affective Events Theory. AET demonstrates how feelings and emotions influence employees' behavior. Affective event theory demonstrates that followers respond emotionally to events and situations they encounter at the workplace and that their response stimulates their wellbeing and satisfaction (23, 48).

Weiss et al. (23) recommend that work events elicit perceptive appraisal and determine emotional reactions, which influence employee outcomes positively or negatively. The work setting shapes an employee's feelings or moods, for example, if they perceive their leader to be less supportive than a colleague. Emotional responses to employees from the past or recent past influence how they feel today. In conclusion, affective event theory gives two key takeaways. First of all, feelings offer important clues to identifying worker behaviors. However, the AET model demonstrates that work set tings' hassles and uplifts affect employee performance and satisfaction. Second, it's important to remember that even seemingly unimportant occurrences can trigger strong emotions in employees. Accordingly, workplace embitterment, a negative emotion, significantly impacts a worker's psychological wellbeing at work. Thus, workplace embitterment, a negative emotion, impact employee wellbeing (24).

**Hypothesis 2:** employee feeling of workplace embitterment is negatively associated with employee wellbeing.

Most studies that looked into the mediating mechanism that starts the connexion between a leader's ethical actions and employee results focus on pro-social elements, such as employee engagement, LMX, and perceived organisational support (18, 49). Few studies looked at how ethical leadership behaviors affect employee wellbeing indirectly through emotional responses, particularly negative emotions in the observed relationship (5, 20, 47, 50). However, this study suggests that by assessing followers' emotional responses during the leadership process, it is possible to indirectly investigate the influence of leaders' ethical behaviors on employee wellbeing. The literature examines employee emotional responses as a mitigator and mediator mechanism. According to Velez and Neves (20), ethical leadership behaviors greatly predict employees' emotional responses and mediate the link between leaders' behaviors and employee outcomes (5, 51). According to a study by Valle et al. (50), an employee's emotional experience reduces the association between a leader's ethical behaviors and the wellbeing of their employees. The study examines workplace embitterment mediating between leaders' ethical behaviors and employees' wellbeing. Therefore, the following theory can be established from the viewpoint of AET.

**Hypothesis 3:** Employee perception of workplace embitterment can mediate the relationship between leaders' ethical behaviors and employees' wellbeing.

### Core self-evaluation: A swapping of leaders' ethical behaviors

Numerous studies in the literature have studied individual attributes and organisational characteristics that can swap the effect of behavior of leader (24). Individual attributes studied as a substitute for leadership behaviors are the locus of control (52) and proactive personality (20). Organisational characteristics include perceived organisational support (53), workplace humour (50), etc. These studies have demonstrated that the presence of any substitutes from individual or organisational characteristics, the less the dependency of an individual on his leader and its influence is reduced [as cited in (20)]. So, aligning with these findings and suggestions, this study expands the literature on leadership swapping. The core self-evaluation attribute of an individual is examined in this study as a potential swapping for ethical leadership behaviors.

Core self-evaluation is an individual evaluation of his abilities, competence, self-worth, and self-control over his environment (51). Individuals with high CSE are less dependent on leaders and more motivated to grasp opportunities and challenges (54). Such individuals are less vulnerable to organisational injustice and leaders' behaviors (20, 55). These individuals' leaders can be redundant; they rely less on their leaders' behaviors. In the scenario (employees with high core self-evaluation) where followers are self-sufficient and less dependent on leaders, it can be assumed that followers' positive core self-evaluation can be swapped or substituted for ethical leadership. Core self-evaluation is an intrinsic motivational resource for coping with environmental challenges and negativity. Conversely, individuals who possess low core self-evaluation experience more negative emotions as they are unable to absorb the negativity from either the work setting or leaders' behaviors. Additionally, these employees are more dependent on their leaders and inept at challenging situations (54).

**Hypothesis 4:** followers with higher CSE experience less workplace embitterment as a result of leaders' low ethical behaviors.

Table 1 summarises all the study hypotheses, their acceptance criteria, and the level of confidence.

**Table 1:** Hypothesis and its acceptance criteria.

**Hypothesis**	**Hypothesis statement**	**Acceptance criteria**	**Confidence interval**
Hypothesis 1	Perceived leaders' ethical behaviors will lower employee exposure to workplace embitterment.	The hypothesis will be accepted if *p* < 0.05	95%
Hypothesis 2	Employee feeling of embitterment is negatively associated with employees' wellbeing	The hypothesis will be accepted if *p* < 0.05	95%
Hypothesis 3	Employee perception of WPE can mediate the relationship between leaders' ethical behaviors (leadership) and employee wellbeing	The hypothesis will be accepted and fully mediates if VAF> 80% and if VAF value < 20% then no mediation	95%
Hypothesis 4	followers with higher CSE experience less workplace embitterment as a result of leaders' low ethical behaviors.	The hypothesis will be accepted if the interaction term is significant (*p* < 0.05). The model will be moderated mediated if the index of moderated mediated is significant.	95%

## Research methods

### Participants and procedure

Participants in the study were faculties at public universities in Pakistan who were recognised by the higher education commission (HEC) as having at least 1 year of professional experience and having interacted with leaders (head of the department). According to ongoing discussions in the *International Journal of Educational Management* (55), because of bullying, abuse, and harassment, modern colleges may not have a favourable work environment for the faculties (55).

A simple random sampling method was used to choose the participants in two stages. In the first stage, a list of all public sector universities was taken from the HEC website, numbered from 1 to n. Then, a sample of 20 universities was selected using MS Excel and between functions. A list of faculties of 20 selected universities was prepared from the faculty profile available on the university website. In the second stage, based on the list of faculties, 800 participants in the study were selected.

A structured questionnaire developed in the English language was used to collect data. Along with the formal questionnaire, an additional sheet was attached explaining the study objective and the clear instructions for the study participants and ensuring the confidentiality of the data. Participants' personal information was kept confidential, and only aggregated responses were used in the study. To lower the common method variance (CMV), as recommended by Podsakoff et al., data were gathered in this investigation in two-time waves separated by 6-week intervals (56).

A link to an online survey-1 is sent to 800 participants. The participants are also being approached *via* phone calls and meeting personally (wherever possible). After receiving the first 570 completed surveys (attrition rate 71%). Six weeks after T1, in period 2 (T2), we send a second survey to T1 respondents, including constructs measuring workplace embitterment and employee wellbeing. We received 411 completed questions on T2 (attrition rate 72%). Table 2 contains information regarding the attrition rate of respondents. Thus, out of the 800 respondents contacted, we found 411 completed, with an average response rate of 73%, which is satisfactory for the two-wave data collection. After removing the outliers, we have 398 responses to work with it.

**Table 2:** Attrition rate of respondents.

**Time lag**	**# of questionnaires delivered**	**# of questionnaires received**	**Attrition rate**
T1	800	570	71%
T2	570	411	72%

Table 3 contains the detailed characteristics of the study sample. The final sample includes 254 male respondents (64%) and 144 female respondents (36%), corresponding to the gender mix of respondents. Most respondents (55%) have an MS/MPhil degree, while the remaining (45%) have a PhD and Post Doctorate. The average age of the respondents was 44.5 years, and their average experience was 11 years.

**Table 3:** Sample characteristics (*n* = 398).

**Category**	**Characteristics**	**% age**
Gender	Male	64
	Female	36
Age	Below 30 years	17
	30–40 years	41
	40–50 years	33
	50–60 years	9
Qualification	Ms/Mphil	55
	PhD	45
	Post Doc	1
Tenure	Below 1 year	3
	1–10 years	58
	10–20 years	12
	20–30 years	24
	Above 30 years	4
Nature of job	Contractual	17
	On BPS	63
	On TTS	20
Job position	Lecturer	48
	Assistant Professor	30
	Associate Professor	19
	Professor	3
Work experience	Below 5 years	35
	5–10 years	46
	10–20 years	14
	20–30 years	4
	Above 30 years	1
Additional assignment	Paid	24
	Unpaid paid	48
	Both	19
	None	8

### Measures

#### Leaders' ethical behaviors (T1)

The ethical leadership scale (α = 0.91) developed by (25) is comprised of different behaviors like fairness, power-sharing, integrity, people orientation, role clarity, and ethical advice, which are used to assess the ethical behavior of leaders. “My HOD discusses what is required of each group member” is an example item. Respondents were asked to rate their responses on a Likert scale with a range of 1–5. Point 1 denotes a strongly disagree, whereas point 5 denotes a strongly agree.

#### Workplace embitterment (T2)

Using the Post-traumatic Embitterment Disorder self-rating scale (α = 0.98) created by Linden et al., workplace embitterment was assessed. The scale (57) has 19 components. The prompt “I have experienced one or more stressful occurrences at work...” appears before each of the 19-item statements. “That I consider being terribly unjust and unfair,” for instance. On a 5-point scale, participants' responses ranged from 1 (not at all true) to 5 (very true).

#### Employee wellbeing (T2)

With nine items and a Likert scale with five possible outcomes, the Zheng et al. (33) scale (α = 0.97) was used to assess the wellbeing of the workforce. Point 1 represents a strongly disagree, whereas Point 5 represents a strongly agree. “People think I'm willing to give and share my time with others” is an example item.

#### Core self-evaluation (T1)

Employees' core self-evaluation is measured by using 12 items scale (α = 0.80) developed by Judge, Erez, Bono, and Thoresen (58) on a Likert scale of 5 points ranging from 1 to 5. Strongly disagree is represented by point 1, and strongly agree by point 5. For instance, “When I fail, I sometimes feel worthless.”

### Control variables

The study controlled for participant gender (coded 1 = male and 2 = female), age (in years), and tenure (in years) with the leader as prior studies, e.g., Schwepker et al. (9) suggested that these characteristics can confound with exposure to workplace embitterment and employee wellbeing.

### Analysis

First, the missing data and outliers are checked and removed using SPSS 24.0 as such cases are potential threats to normal data distribution. Second, the measurement model requirements such as confirmatory factor analysis of all constructs are investigated using AMOS 24.0. Then, the reliability and validity detail investigation is done by measuring composite reliability and convergent and divergent validity of the measurement model. Hair et al. (59) suggested that the construct-related standardised weights calculated the average variance scores. Third, common method bias, also a serious concern of the study, is investigated using Harman's single factor score, wherein all items (measuring unobserved variables) stay loaded into one common measure. The overall variance for one (single) factor must be lower than 50%, and if the overall variance is lower than 50%, it is an indication that common method bias would not affect the data and the results (56). Next, descriptive statistics, correlation analysis, and Cronbach alphas of all constructs are estimated using SPSS 24.0. Lastly, hypotheses testing is completed on Hayes Process Macro (2017) using a bias confidence interval.

## Results

Table 4 presents the descriptive statistics, including mean and standard deviation and associations between the study constructs. According to correlation coefficients listed in Table 4, ethical leadership behavior is strongly and negatively related to workplace embitterment (*r* = −0.454, *p* = 0.01). Employees who believe their leaders to be morally upright report less embitterment at work. WPE and employee wellbeing are strongly and adversely correlated (*r* = −0.759, *p* = 0.01). Thus, perceived workplace embitterment significantly and negatively influences employees' wellbeing. Moreover, all correlations were significant and associated at a moderate level, indicating no multicollinearity issue (60). This gave the premise for further testing of study hypotheses.

**Table 4:** Descriptive statistics and inter-correlations of study variables.

**Variable**	**CR**	**AVE**	**Mean**	**SD**	**1**	**2**	**3**	**4**
1. Leaders ethical behaviors	0.85	0.51	2.54	0.52	**0.708**			
2. Workplace embitterment	0.96	0.87	3.16	1.12	−0.454	**0.933**		
3. Employee wellbeing	0.97	0.92	2.65	1.08	0.378	−0.759	**0.959**	
4. Core self-evaluation	0.83	0.58	2.89	0.57	−0.249	−0.303	0.157	**0.762**

CR, Composite reliability; AVE, Average variance extracted; p < 0.01. Bold values indicate the square root of every construct AVE to determine discriminant validity.

### Measurement model

The validity of each study construct is estimated using confirmatory factor analysis. This study's constructs comprise many item scales (e.g., workplace embitterment 19 items). So, the item parcelling approach is adopted to avoid the problem of model under-identification. Due to items parcelling, a small number of parameters fetches stable scales, trivial standard errors, and a better model fit (61). The items allotted to every parcel were averaged. Thus, a minimum of 3 parcels are formed for every construct of the study: the leader's ethical behaviors, workplace embitterment, employee wellbeing and core self-evaluation. The CFA results show that all variables are independent of each other. Chi-square (χ^2^) = 280.261, χ^2^ / degrees of freedom (df) = 2.860, *p* < 0.000, comparative fit index (CFI) = 0.968, [Tucker – Lewis index (TLI)] = 0.961, The goodness of fit index (GFI) = 0.918, root-mean-square error approximation (RMSEA) = 0.06, and standardised root means residual (SRMR) = 0.043. All values are within acceptable ranges (62).

Moreover, Cronbach's alpha (0.76–0.98) and composite reliability (CR) values (0.803–0.97) are all above the threshold of 0.70; and AVE values (0.51–0.921) are all comfortably above the threshold of 0.50. The measurement model's convergent validity is guaranteed by the alpha, composite reliability, and average variance extracted values (see Table 4). The value of each variable's square root should be over the various inter-construct correlations, according to Fornell and Larcker's (63) criterion, which is used to assess discriminant validity. On the diagonals of Table 4, the square root of the AVE of each observable variable is given. On in-depth examination of those figures, the square root of the AVE of each variable is shown to have the highest connexion with other variables of each given variable. Thus, it confirmed the discriminant validity.

Due to the limitations of the testing of one Harman feature, Podsakoff et al. (64), the mensuration model is tested with and while not a common latent factor (CLF) to evaluate the extent to that common method bias (CMB) may be a major data issue. A common latent factor may be a hidden feature within the mensuration model that features a direct relationship with the models' all variables (constructs). A different mensuration model is run, containing a common latent factor with direct paths to any or all indicators of all constructs of the mensuration model. CLF variance is restricted to 1 [as cited in (65)]. CLF measurement model fit values (χ^2^ =279.729, *p* < 0.01; χ^2^ / DF =2.741; RMSEA = 0.061; SRMR 0.0470; NFI is 0.92; GFI is 0.89; TLI is 0.94; CFI is 0.95) reported a good model fit.

### Common method bias

The difference of standardised regression weights of the measurement model without CLF and with CLF is calculated to cheque the degree of CMB, and none of the individual differences is >0.2, which reported that CMB threat is not found in the data (65). Moreover, the explained variance by the common factor method is only 4%, far from the threshold value is 25% [as cited in (53)].

### Hypotheses testing

**Figure 2:**
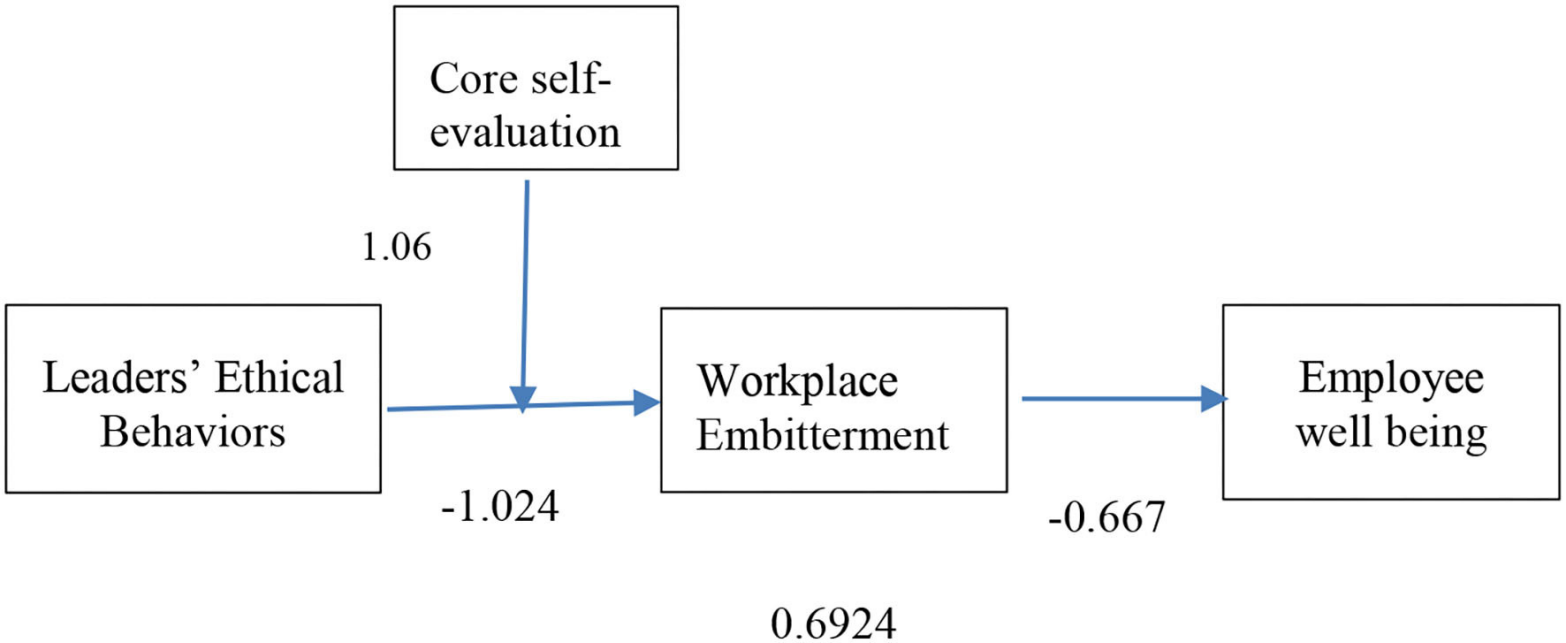
Structural model with path estimates.

The study's hypotheses are tested using Hayes' (66) process macro for SPSS, and the findings are shown in Table 5. These results indicate the existence of a significant relationship between studied variables, and almost all results are aligned with findings in the literature. First, we examined the direct relationships, and then the indirect (mediation) relationships were examined. The outline of the results is shown in Figure 2.

**Table 5:** Mediation model's path coefficients and indirect effects.

**Structural path**	**β**	**SE**	***P*-value**	**95% CI**	
				**Lower limit**	**Upper limit**
LEB-EWB	0.1011	0.0793	0.2031	−0.0548	0.257
LEB-WPE	−1.0224^*^	0.0956	0.000	−1.2014	−0.8343
WPE-EWB	−0.6673^*^	0.0367	0.000	−0.7494	−0.6051
LEB-WPE-EWB	0.6924^*^	0.0696	0.000	0.5597	0.8335
Total effect	0.7935	0.0952	0.000	0.6064	0.9807
**Control variables**		β	**SE**		***P*-value**
Age		0.058	0.063		0.353
Gender		0.187	0.109		0.09
Tenure with current leader		0.037	0.076		0.627

N = 398,

* *p* < 0.01.

### Direct relationships

The estimation of the study relationships is shown in Figure 2. According to hypothesis 1, ethical behavior on the part of leaders is negatively correlated with workplace embitterment, and according to hypothesis 2, WPE and employee wellbeing are negatively correlated. The results also show that ethical behaviors in leaders (β = – 1.024, *p* < 0.01) is significantly negatively related to WPE, in support of H1 and WPE (β = – 0.667, *p* < 0.01) negatively related to employee wellbeing, in support of H2.

### Indirect relationships (testing of mediation and moderation)

Mediation (indirect relationship) between predicting and outcome variables exist when with any change in predicting variable, the mediating variable also changes, which subsequently affects the outcome variable (59). Hypothesis 3 proposes that employee perception of WPE can mediate the association between LEBs and employee wellbeing.

Hayes' (66) macro process results measuring the indirect relationship (mediation) between LEBs and employees' wellbeing are given in Table 5. According to the findings, there is a significant indirect impact of LEBs (through WPE) on employee wellbeing [= 0.6924, *p* = 0.001, 95% CI (0.5560, 0.8372)]. Furthermore, the analysis of bias-corrected bootstrap exposes that the 95% confidence interval (CI) mentioned above excludes 0 for the mediating effect of workplace embitterment, supporting H3. Variance accounted for (VAF), which cheques the partial or full mediation effect. VAF is a ratio of indirect effect to total effect (total effect = indirect effect + direct effect). The value of VAF for WPE in the leaders' ethical behaviors and employee wellbeing relationship is 0.87 (87%), indicating full mediation (67). Moreover, the model direct effects revealed that ethical leadership (β = 0.1011, 95% CI (−0.0367, 0.1694)] is not significant and which also indicates that WPE fully mediates the relationship between leader's ethical behaviors, such as being fair, truthful, delegating power, people orientation, clarifying the roles to followers, and guiding them ethically, and employee wellbeing.

### Testing of moderated mediation

According to Hypothesis 4, employee core self-evaluation moderates the negative association between leaders' ethical behaviors and workplace embitterment, making it lesser in the presence of high core self-evaluation and stronger if employees gave their CSE a lower rating. The findings of this hypothesis analysis are presented in Table 6, which uses Hayes' (66) process macro moderated mediated model, also known as model 7.

**Table 6:** Regression results for the conditional indirect effect.

**Structural path**	**β**	**SE**	**95% CI**	
			**Lower limit**	**Upper limit**
LEB-WPE	−4.1665^**^	0.4017	−4.9563	−3.367
LEB-EWB	0.1011	0.0793	−0.0548	0.2570
CSE-WPE	−3.3274^**^	0.3629	−4.0408	−2.1639
ELS ^*^ CSE-WPE	1.0616^**^	0.1381	0.7900	1.331
**Index of moderated mediation**				
CSE as moderator	−0.719^*^	0.0947	−0.909	−0.5307
Conditions of the moderator	Indirect effect	Standard error	95% CI	
Low (M-1SD)	1.0844	0.0906	0.913	1.2714
Medium (M+0SD)	0.7294	0.07	0.5893	0.8653
High (M+1SD)	0.3654	0.0779	0.2084,	0.5174

* *p* < 0.05;

** *p* < 0.01.

The upper parts of Table 6 contain the coefficient of the moderation effect of CSE on the negative link between LEBs and workplace embitterment, which indicates that the interaction term resulted by multiplying CSE and leaders' ethical behaviors (leadership; β = 1.0616, *p* < 0.01), is significant.

Next, we perform a basic slope analysis (66). First, we looked at how employee core self-evaluation and leaders' ethical behaviors (leadership) interacted to affect workplace embitterment. The moderated mediation index [index = 0.719, SE = 0.0947, CI (−0.9090, −0.5307)] is significant. Additionally, conditional indirect effects are seen in Table 6's lower sections at various points, with one minus SD and one plus SD. The results show that the mediated model for leaders' ethical behaviors (IV) is significant when employee core self-evaluation is high (i.e., one standard deviation above the mean). The conditional indirect effect is = 0.3654, SE is 0.0779, and the confidence interval is (0.2084, 0.5174).

**Figure 3:**
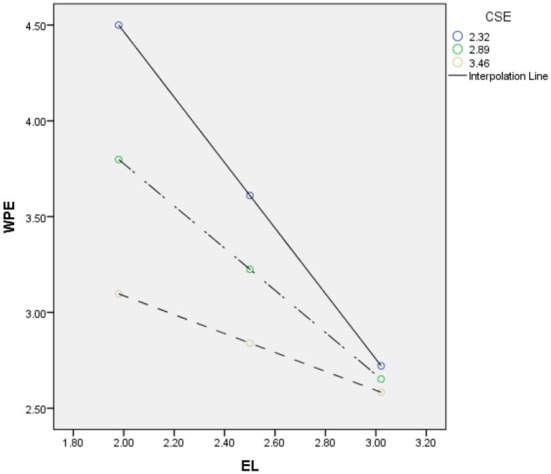
Regression results for the conditional indirect effect.

When employee core self-evaluation is low (i.e., 1 standard deviation below the mean), the mediated model for leaders' ethical behaviors (leadership) is significant. The conditional indirect effect is = 1.0844, SE = 0.0906, CI (0.9130, 1.2714). Overall, the pattern shown in Figure 2 supports H4. That is, leaders' low ethical behaviors are linked to lower employee wellbeing through intensified employee emotion, i.e., workplace embitterment, but only when employee core self-evaluation is low (see Figure 3). Likewise, when employees have a higher perception of their core self-evaluation, low leaders' ethical behaviors are related to improved or stable wellbeing through the decreased effect of negative emotion of employee, i.e., workplace embitterment (i.e., swapping of ethical leadership).

## Discussion

This study focused on the research question of how leaders' ethical behaviors mend employees' wellbeing by precluding employees' negative emotions at work. Numerous studies on leaders' ethical behaviors (ethical leadership) demonstrated that LEBs affect employees' behaviors and outcomes (20, 49, 55). Employees perceive their leaders' behaviors as ethical due to the features of being supported by leaders, being treated fairly and justice, and sharing the powers. Therefore, they experience fewer negative events and negative emotions, which foster their wellbeing.

### Research results

This study proposed negative workplace emotion (workplace embitterment) as a mediation mechanism in leader's ethical behaviors and employee wellbeing relationships based on affective events theory in the context of Ahmad and Kaleem's study concluded that discuss the positive link between leaders' ethical behaviors and employees' wellbeing (55). Due to the unpleasant affective experiences (embitterment) at work, leaders' unfair and unethical activities were observed to have an adverse effect on employee wellbeing in the study context (a collectivist culture) where individuals feel obligated to favour their close ones (55). The study's findings are based on the data gathered from Pakistan's public universities' faculties. Primarily, it was found that LEBs will help in preventing negative experiences and emotions in the workplace, which will nourish employees' wellbeing. Furthermore, this study investigates WPE as a mediation mechanism in leaders' ethical behaviors and employee wellbeing relationships and found a fully mediating role. Mainly, it catechises the role of leaders' (un)ethical behaviors in cropping followers' emotional reactions to employee wellbeing (5, 20, 55). The study derives leaders' ethical behaviors enhance followers' wellbeing by minimising the chances of emergence of negative emotion in leader-follower daily interactions.

Generally, noting the dearth of an investigation into the influence of leaders' ethical behavior on inducing emotional reactions, this study validates the hypothesis that leaders' ethical behaviors can restrain workplace embitterment. Leaders' ethical behaviors are negatively associated with workplace embitterment, whether in the shape of PTSD, bullying, or stress disorder, in line with the literature [e.g., (19, 20, 44, 45)]. Moreover, the study's results also divulge that employee wellbeing is smashed due to workplace embitterment, consistent with prior studies (2, 7, 19, 47, 48, 68, 69). An interesting finding of this study is demonstrating how a leader's ethical behaviors play an influential role in eluding negative emotions within the work and nourishing employee wellbeing, which is imperative for every organisation.

This study also investigates a catalyst that can swap the effect of low leaders' ethical behaviors in the organisation (16, 55). Followers' CSE is a moderator in the study relationship, which has a dual impact. On one side, it makes followers independent of leaders; on the other, it maintains followers' wellbeing by breaking down the impacts of leaders' low ethical behaviors on WPE (20). Employees with high CSE always do things better due to their abilities and perception of control over the changing situation (54). Research on employees with high CSE has suggested that these employees possess the flexibility and competence to deal with situational challenges (54). Therefore, employees with high CSE have greater social and personal self-efficacy and locus of control in slashing the effects of negative work events.

This study expands the scope of appraisal theories of emotions by elucidating leaders' ethical behaviors as a stimulant of employee wellbeing by controlling WPE. Moreover, this study also implies that workplace embitterment mediates the link between a leader's ethical behaviors and employees' wellbeing, as suggested by numerous researchers (20, 50, 68–70). It is alluring that employees with high core self-evaluation are likelier to be independent of leaders' ethical behaviors and potentially swap the low leader's ethical behaviors.

### Theoretical contributions

This study has valued and is worth citing strengths. First and foremost, this study is significant because it adds to the literature on employee wellbeing and employee emotions by organisational (leaders' behaviors) aspect as well as follower traits, such as core self-evaluation. Explicitly, there is a paucity of literature on workplace embitterment, particularly in terms of leader behaviors and followers' emotional experiences during the leadership process. The purpose of this study is to see how leaders' ethical behaviors, as a contextual variable, affect employee wellbeing by restraining negative emotion, i.e., workplace embitterment, from the AET perspective. The study underlines the importance of leaders' ethical behavior in developing countries, as well as a collectivist culture, in encouraging employee wellbeing by preventing workplace embitterment. It aids in seeing the logical link between a leader's ethical behaviors and unfavourable employee emotions, such as workplace embitterment. Therefore, from the perspective of AET, this study confirms that workplace embitterment which is a negative emotion serves as a mediating mechanism by which leaders' ethical behaviors affect employees' wellbeing. It also affirms the significance of leaders' ethical behaviors in managing negative emotions and promoting employee wellbeing.

The second strength of the study is the exhaustive examination of leaders' ethical behaviors to embitterment and employee wellbeing relationships from a different theoretical perspective. Unlike other studies in the literature based on social exchange, ethical leadership, and social learning theories, this study is based on appraisal theories of emotions.

Finally, concerning the limited literature on the handling of workplace negative emotions, i.e., embitterment, this study adds to the knowledge by suggesting follower traits, such as CSE, as an alternative to prevent or reduce workplace embitterment and improve employee wellbeing. This study also contributes to a growing area of research concerned with emotional management in the workplace where low leader's ethical behavior (ineffective leadership) is dominating and effective leadership is ignored by examining the role of employee's characteristics, such as core self-evaluation, which may mitigate the effect of ineffective leadership. Thus, the findings of this study emphasise the effectiveness of followers' core self-evaluation trait in managing their emotions when leaders practice low ethical behaviors, and such practices are prevalent. Thus, its findings suggest that followers CSE swap leaders' ethical behaviors as an alternative mechanism to foster employee wellbeing.

### Practical implications

First and foremost, several studies in the literature demonstrated that leaders' ethical behaviors and ethical leadership are one of the significant factors affecting employees' imperative behaviors and wellbeing positively (28–30, 69, 70). This study considered the affective perspective, which potentially affects the relationship between leaders' ethical behaviors and employees' wellbeing. Our study findings confirmed that leaders' ethical behaviors nourish employees' wellbeing by avoiding the emergence of negative emotion, i.e., workplace embitterment. So, this study provides an opportunity for the management to understand the vitality of leaders' ethical behaviors in averting employees from being embittered and taming wellbeing. Hence, it provides a baseline for longitudinal tracking of changes in leaders' behaviors, such as developing transparent or ethical work culture and training programs for leaders to be ethical, constituting clear ethical guidelines, and practicing by leaders who impel followers' emotions and behaviors.

Second, our study findings confirmed the eminence of leaders' ethical behaviors to develop positive behaviors and prevent negative emotions. Mostly, organizations in the contemporary business world prefer competence and performance while selecting leaders owing to high competition and uncertainties in the market. However, less consideration is given to morality and ethical attributes to leaders in a unique position to influence their followers. Therefore, this study guides organizations to ensure that the right person is hired or promoted as a leader or manager that can behave ethically and maintain an ethical work environment.

Finally, though it's hard to ensure that our leaders behave ethically, this accentuates some alternatives that can substitute leaders' ethical behaviors or reduce dependency on leaders. Literature on leaders' ethical behaviors explains attributes that make leaders either unnecessary or less dependent on leaders [e.g., (20)]. Our study findings supported that followers' attributes, such as CSE, can moderate the link between leaders' ethical behaviors and followers' negative emotions, i.e., workplace embitterment, and can swap leaders' ethical behaviors. Therefore, leaders' organizations should pay attention to the personality attributes of employees during the selection process. They should prefer employees who rate higher on core self-evaluation attributes. Simultaneously, organizations should continually make efforts to raise their core self-evaluation attribute through training programs, feedback, reward system, etc.

### Limitations and novel future directions

The first limitation is common method variance (CMV) occurrence since employees provided ratings of leaders' ethical behaviors, workplace embitterment, employee wellbeing, and core self-evaluation. However, to abolish CMV and to obtain more worthwhile results, data should be collected in dyadic (56). Data collected in dyadic provides information closer to reality as dyadic data collection is free of personal over or under estimations. Second, this research work adopts a study design based on time interval, which is better than the cross-sectional study design. However, it's less accessible than experimental and longitudinal research designs. Future research can use such designs to formalise the cause as these study designs are viable and provide the opportunity to highlight some potential exceptional.

Third, this study has found that followers' CSE is a moderator between leaders' ethical behaviors and WPE. Other personality traits such as emotional stability and general self-efficacy may be examined as a swap of leaders' ethical behaviors (71, 72). Finally, the study is context specific and conducted in one country, Pakistan; study results may vary in different countries as LEB practice may be more prominent in the individualistic culture where people behave rationally than emotionally, and the chance of being unfair, dealing with inhumation is low (55). Yet without formal cultural testing features, this estimate is very speculative, so we recommend further research explicitly examining culture's role in the observed relationship.

## Conclusion

The relationship between leaders' ethical behaviors and employees' wellbeing has been examined widely, but most studies examine positive and relational aspects. Stunningly, limited studies have examined the affective perspective. Based on appraisal theories of emotions, the study results suggest that followers' workplace embitterment completely mediates the relationship between leaders' ethical behaviors and employees' wellbeing. Moreover, followers' perceptions of high core self-evaluation can moderate the observed relationship and may swap leaders' ethical behaviors. However, this study validates (i) Further, this study identifies the ethical areas which need attention from the leaders to sway employee behavior; (ii) the significant role of a leader's ethical behaviors in nourishing employee wellbeing by preventing negative emotions; (iii) management have to take proactive actions such as devising clear code of conduct and communicating to leaders, ensure hiring of leaders with strong ethical values, reward ethical behaviors of leaders to encourage LEB in the organisation, etc.; and (iv) it also establishes the role of core self-evaluation in swapping leader's ethical behaviors. Although there is still more work to be done, we hope that our study will inspire other researchers to advance our understanding of leaders' ethical behaviors and employee wellbeing.

## Data availability statement

The raw data supporting the conclusions of this article will be made available by the authors, without undue reservation.

## Author contributions

AS and MB developed the research idea, design, and methodology. AS and MA conducted data collection and revised the manuscript. AS conducted the data analysis, prepared the original draft, and interacted with reviewers and editor. AS, MA, and MB approved and reviewed the article's submission. All authors contributed to the article and approved the submitted version.

## Conflict of interest

The authors declare that the research was conducted in the absence of any commercial or financial relationships that could be construed as a potential conflict of interest.

## Publisher's note

All claims expressed in this article are solely those of the authors and do not necessarily represent those of their affiliated organizations, or those of the publisher, the editors and the reviewers. Any product that may be evaluated in this article, or claim that may be made by its manufacturer, is not guaranteed or endorsed by the publisher.

## References

[1] Rudolph CW Breevaart K Zacher H. Disentangling between-person and reciprocal within-person relations among perceived leadership and employee well-being. J Occup Health Psychol. (2022) 2022:3cdeg. 10.31234/osf.io/3cdeg 35175081

[2] Zhang Z Wang J Jia M. Multilevel examination of how and when socially responsible human resource management improves the well-being of employees. J Bus Ethics. (2022) 176:55–71. 10.1007/s10551-020-04700-4

[3] Glasø L Notelaers G. Workplace bullying, emotions, and outcomes. Violence Vict. (2012) 27:360–77. 10.1891/0886-6708.27.3.360 22852437

[4] Carter C. Organisational injustice in UK frontline services and the onset of Moral Injury, Post Traumatic Embitterment Disorder (PTED) and PTSD. Int J Law Crime Justice. (2021) 66:100483. 10.1016/j.ijlcj.2021.100483

[5] Schwepker CH Jr Dimitriou CK. Using ethical leadership to reduce job stress and improve performance quality in the hospitality industry. Int J Hospital Manag. (2021) 94:102860. 10.1016/j.ijhm.2021.102860

[6] Michailidis E. Exploring the Feeling of Embitterment in the Workplace. Guildford: University of Surrey (2017).

[7] Dey M Bhattacharjee S Mahmood M Uddin MA Biswas SR. Ethical leadership for better sustainable performance: role of employee values, behavior and ethical climate. J Clean Prod. (2022) 337:130527. 10.1016/j.jclepro.2022.130527

[8] Schmidt S Tinti C Levine LJ Testa S. Appraisals, emotions and emotion regulation: an integrative approach. Motiv Emot. (2010) 34:63–72. 10.1007/s11031-010-9155-z 20376165 PMC2844958

[9] Schwepker CH Valentine SR Giacalone RA Promislo M. Good barrels yield healthy apples: organisational ethics as a mechanism for mitigating work-related stress and promoting employee well-being. J Bus Ethics. (2021) 174:143–59. 10.1007/s10551-020-04562-w

[10] Giacalone RA Promislo MD. Handbook of Unethical Work Behavior: Implications for Individual Well-Being: Implications for Individual Well-Being. London: Routledge (2014). 10.4324/9781315703848

[11] Brown ME Mitchell MS. Ethical and unethical leadership: exploring new avenues for future research. Bus Ethics Quarterly. (2010) 20:583–616. 10.5840/beq201020439

[12] Brown ME Treviño LK Harrison DA. Ethical leadership: A social learning perspective for construct development and testing. Organ Behav Hum Decis Process. (2005) 97:117–34. 10.1016/j.obhdp.2005.03.002

[13] Sarwar A Naseer S Zhong JY. Effects of bullying on job insecurity and deviant behaviors in nurses: roles of resilience and support. J Nurs Manag. (2020) 28:267–76. 10.1111/jonm.12917 31788904

[14] Resick CJ Martin GS Keating MA Dickson MW Kwan HK Peng C. What ethical leadership means to me: Asian, American, and European perspectives. J Bus Ethics. (2011) 101:435–57. 10.1007/s10551-010-0730-8

[15] Ahmed OM Kamil BA Ishak AK. Influence of perceived stress and organisational justice on employee wellbeing amongst academia: a conceptual paper. Int J Acad Res Bus Soc Sci. (2018) 8:396–409. 10.6007/IJARBSS/v8-i8/4477

[16] Zhu W He H Treviño LK Chao MM Wang W. Ethical leadership and follower voice and performance: the role of follower identifications and entity morality beliefs. Leadersh Quart. (2015) 26:702–18. 10.1016/j.leaqua.2015.01.004

[17] Hassan S Mahsud R Yukl G Prussia GE. Ethical and empowering leadership and leader effectiveness. J Manag Psychol. (2013). 10.1108/02683941311300252

[18] Den Hartog DN Belschak FD. Work engagement and Machiavellianism in the ethical leadership process. J Bus Ethics. (2012) 107:35–47. 10.1007/s10551-012-1296-4

[19] Michailidis E Cropley M. Testing the benefits of expressive writing for workplace embitterment: a randomised control trial. Eur J Work Org Psychol. (2019) 28:315–28. 10.1080/1359432X.2019.1580694

[20] Velez MJ Neves P. Shaping emotional reactions to ethical behaviors: proactive personality as a substitute for ethical leadership. Leadersh Quart. (2018) 29:663–73. 10.1016/j.leaqua.2018.06.004

[21] Zheng D Witt LA Waite E David EM van Driel M McDonald DP. Effects of ethical leadership on emotional exhaustion in high moral intensity situations. Leadersh Quart. (2015) 26:732–48. 10.1016/j.leaqua.2015.01.006

[22] Strom DL Sears KL Kelly KM. Work engagement: the roles of organisational justice and leadership style in predicting engagement among employees. J Leadersh Org Stud. (2014) 21:71–82. 10.1177/1548051813485437

[23] Weiss HM Suckow K Cropanzano R. Effects of justice conditions on discrete emotions. J Appl Psychol. (1999) 84:786. 10.1037/0021-9010.84.5.78626651622

[24] Lazarus RS Kanner AD Folkman S. Emotions: a cognitive–phenomenological analysis. In: Theories of Emotion. Cambridge, MA: Academic Press (1980). p. 189–217. 10.1016/B978-0-12-558701-3.50014-4

[25] Kalshoven K Den Hartog DN De Hoogh AH. Ethical leadership at work questionnaire (ELW): Development and validation of a multidimensional measure. Leadersh Quart. (2011) 22:51–69. 10.1016/j.leaqua.2010.12.007

[26] Northouse. PG: Introduction to Leadership: Concepts and Practice. Thousand Oaks, CA: SAGE Publications, Incorporated (2014). p. 352.

[27] Watts T. Business Leaders' Values Beliefs Regarding Decision Making Ethics. Lulu.com (2008).

[28] Bello SM. Impact of ethical leadership on employee job performance. Int J Bus Soc Sci. (2012) 3. 10.30845/ijbss

[29] Çelik S Dedeoglu BB Inanir A. Relationship between ethical leadership, organisational commitment and job satisfaction at hotel organizations. Ege Acad Rev. (2015) 15:53–64. 10.21121/eab.2015117999

[30] Ma Y Cheng W Ribbens BA Zhou J. Linking ethical leadership to employee creativity: knowledge sharing and self-efficacy as mediators. Soc Behav Personal. (2013) 41:1409–19. 10.2224/sbp.2013.41.9.1409

[31] Dinc MS Aydemir M. Ethical leadership and employee behaviors: an empirical study of mediating factors. Int J Bus Govern Ethics. (2014) 9:293–312. 10.1504/IJBGE.2014.064738

[32] Dinc MS. Direct and indirect effect of ethical leadership on employee behaviors in higher education. Int J Manag Educ. (2018) 12:201–22. 10.1504/IJMIE.2018.10012319

[33] Zheng X Zhu W Zhao H Zhang C. Employee well-being in organizations: theoretical model, scale development, and cross-cultural validation. J Organ Behav. (2015) 36:621–44. 10.1002/job.1990

[34] Larsen RJ Diener ED Emmons RA. An evaluation of subjective well-being measures. Soc Indic Res. (1985) 17:1–7. 10.1007/BF00354108

[35] Ryff CD Keyes CL. The structure of psychological well-being revisited. J Pers Soc Psychol. (1995) 69:719. 10.1037/0022-3514.69.4.719 7473027

[36] Chen FF Jing Y Hayes A Lee JM. Two concepts or two approaches? A bifactor analysis of psychological and subjective well-being. J Hap Stud. (2013) 14:1033–68. 10.1007/s10902-012-9367-x

[37] Page KM Vella-Brodrick DA. The ‘what',‘why'and ‘how'of employee well-being: a new model. Soc Indic Res. (2009) 90:441–58. 10.1007/s11205-008-9270-3

[38] Bedi A Alpaslan CM Green S. A meta-analytic review of ethical leadership outcomes and moderators. J Bus Ethics. (2016) 139:517–36. 10.1007/s10551-015-2625-1

[39] Gilbreath B Benson PG. The contribution of supervisor behavior to employee psychological well-being. Work Stress. (2004) 18:255–66. 10.1080/02678370412331317499

[40] Inceoglu I Thomas G Chu C Plans D Gerbasi A. Leadership behavior and employee well-being: an integrated review and a future research agenda. Leadersh Quart. (2018) 29:179–202. 10.1016/j.leaqua.2017.12.006

[41] Abdelmotaleb M Metwally A Saha SK. Servant leadership and nurses' upward voice behavior in an Egyptian hospital: Does prosocial motivation matter? Hum Syst Manag. (2022) 41:47–58. 10.3233/HSM-201134

[42] Linden M. Posttraumatic embitterment disorder. Psychother Psychosom. (2003) 72:195–202. 10.1159/000070783 12792124

[43] Sensky T. Chronic embitterment and organisational justice. Psychother Psychosom. (2010) 79:65–72. 10.1159/000270914 20051704

[44] Muschalla B Linden M. Embitterment and the workplace. In: Embitterment. Vienna: Springer (2011). p. 154–67. 10.1007/978-3-211-99741-3_12

[45] Zhou H Sheng X He Y Qian X. Ethical leadership as the reliever of frontline service employees' emotional exhaustion: a moderated mediation model. Int J Environ Res Public Health. (2020) 17:976. 10.3390/ijerph17030976 32033237 PMC7037031

[46] Bono JE Foldes HJ Vinson G Muros JP. Workplace emotions: the role of supervision and leadership. J Appl Psychol. (2007) 92:1357. 10.1037/0021-9010.92.5.1357 17845090

[47] Aghighi A. The role of ethical leadership and proactive personality on organizational citizenship behaviors: explaining the mediating effect of positive and negative emotions. Int J Ethics Soc. (2020) 2:11–8. Available in online: http://ijethics.com/article-1-77-en.html

[48] Glomb TM Steel PD Arvey RD. Office sneers, snipes, and stab wounds: antecedents, consequences, and implications of workplace violence and aggression. Emot Workplace. (2002) 227:259.

[49] Loi R Lam LW Ngo HY Cheong SI. Exchange mechanisms between ethical leadership and affective commitment. J Manag Psychol. (2015) 2013:278. 10.1108/JMP-08-2013-0278

[50] Valle M Kacmar M Andrews M. Ethical leadership, frustration, and humor: a moderated-mediation model. Leadersh Org Dev J. (2018) 2018:83. 10.1108/LODJ-02-2018-0083

[51] Kim M Beehr TA. Job crafting mediates how empowering leadership and employees' core self-evaluations predict favourable and unfavourable outcomes. Eur J Work Org. Psychol. (2020) 29:126–39. 10.1080/1359432X.2019.1697237

[52] De Hoogh AH Den Hartog DN. Neuroticism and locus of control as moderators of the relationships of charismatic and autocratic leadership with burnout. J Appl Psychol. (2009) 94:1058. 10.1037/a0016253 19594244

[53] Rineer JR Truxillo DM Bodner TE Hammer LB Kraner MA. The moderating effect of perceived organisational support on the relationships between organisational justice and objective measures of cardiovascular health. Eur J Work Org Psychol. (2017) 26:399–410. 10.1080/1359432X.2016.1277207

[54] Nevicka B Van Vianen AE De Hoogh AH Voorn B. Narcissistic leaders: an asset or a liability? Leader visibility, follower responses, and group-level absenteeism. J Appl Psychol. (2018) 103:703. 10.1037/apl0000298 29553765

[55] Ahmad S Fazal-E-Hasan SM Kaleem A. How ethical leadership stimulates academics' retention in universities: the mediating role of job-related affective well-being. Int J Educ Manag. (2018) 2017:324. 10.1108/IJEM-11-2017-0324

[56] Podsakoff PM MacKenzie SB Lee JY Podsakoff NP. Common method biases in behavioral research: a critical review of the literature and recommended remedies. J Appl Psychol. (2003) 88:879. 10.1037/0021-9010.88.5.879 14516251

[57] Linden M Baumann K Lieberei B Rotter M. The post-traumatic embitterment disorder self-rating scale (PTED scale). Clin Psychol Psychother. (2009) 16:139–47. 10.1002/cpp.610 19229838

[58] Judge TA Erez A Bono JE Thoresen CJ. The core self-evaluations scale: development of a measure. Pers Psychol. (2003) 56:303–31. 10.1111/j.1744-6570.2003.tb00152.x

[59] Hair JF Black WC Babin BJ Anderson RE Tatham RL. Multivariate data analysis. Upper Saddle River. (1998) 5:207–19.

[60] Wheeler D Tiefelsdorf M. Multicollinearity and correlation among local regression coefficients in geographically weighted regression. J Geogr Syst. (2005) 7:161–87. 10.1007/s10109-005-0155-6

[61] Bandalos DL. The effects of item parceling on goodness-of-fit and parameter estimate bias in structural equation modeling. Struct Equ Model. (2002) 9:78–102. 10.1207/S15328007SEM0901_5

[62] Anderson JC Gerbing DW. Structural equation modeling in practice: a review and recommended two-step approach. Psychol Bull. (1988) 103:411. 10.1037/0033-2909.103.3.411

[63] Fornell C Larcker DF. Structural Equation Models With Unobservable Variables and Measurement Error: Algebra and Statistics.

[64] Podsakoff PM MacKenzie SB Podsakoff NP. Sources of method bias in social science research and recommendations on how to control it. Annu Rev Psychol. (2012) 63:539–69. 10.1146/annurev-psych-120710-100452 21838546

[65] Lowry PB Gaskin J. Partial least squares (PLS) structural equation modeling (SEM) for building and testing behavioral causal theory: when to choose it and how to use it. IEEE Trans Prof Commun. (2014) 57:123–46. 10.1109/TPC.2014.2312452

[66] Hayes AF Montoya AK Rockwood NJ. The analysis of mechanisms and their contingencies: PROCESS versus structural equation modeling. Aust Market J. (2017) 25:76–81. 10.1016/j.ausmj.2017.02.001

[67] Hair JF Jr Sarstedt M Hopkins L Kuppelwieser VG. Partial least squares structural equation modeling (PLS-SEM): an emerging tool in business research. Eur Bus Rev. (2014). 10.1108/EBR-10-2013-0128

[68] Doty DH Glick WH. Common methods bias: does common methods variance really bias results? Org Res Methods. (1998) 1:374–406. 10.1177/109442819814002

[69] Ahmad S Sohal AS Cox JW. Leading well is not enough: a new insight from the ethical leadership, workplace bullying and employee well-being relationships. Eur Bus Rev. (2020) 2018:149. 10.1108/EBR-08-2018-0149

[70] Hassan S. We need more research on unethical leadership behavior in public organizations. Public Integr. (2019) 21:553–6. 10.1080/10999922.2019.1667666

[71] Judge TA Kammeyer-Mueller JD. Implications of core self-evaluations for a changing organisational context. Hum Resour Manag Rev. (2011) 21:331–41. 10.1016/j.hrmr.2010.10.003

[72] Ilyas S Abid G Ashfaq F. Ethical leadership in sustainable organizations: the moderating role of general self-efficacy and the mediating role of organisational trust. Sustain Prod Consumpt. (2020) 22:195–204. 10.1016/j.spc.2020.03.003

